# A Nationwide Registry-Based Study on Mortality Due to Rare Congenital Anomalies

**DOI:** 10.3390/ijerph15081715

**Published:** 2018-08-10

**Authors:** Verónica Alonso-Ferreira, Germán Sánchez-Díaz, Ana Villaverde-Hueso, Manuel Posada de la Paz, Eva Bermejo-Sánchez

**Affiliations:** 1 Institute of Rare Diseases Research (IIER), Instituto de Salud Carlos III & Centre for Biomedical Network Research on Rare Diseases (CIBERER), 28029 Madrid, Spain; anavillaverde@isciii.es (A.V.-H.); mposada@isciii.es (M.P.d.l.P.); eva.bermejo@isciii.es (E.B.-S.); 2 Institute of Rare Diseases Research (IIER), Instituto de Salud Carlos III & Department of Geology, Geography and Environmental Sciences, University of Alcala (UAH), 28801 Madrid, Spain; g.sanchez@externos.isciii.es

**Keywords:** rare diseases, congenital anomalies, population-based mortality, geographical analysis, time trend

## Abstract

This study aimed to analyse population-based mortality attributed to rare congenital anomalies (CAs) and assess the associated time trends and geographical differences in Spain. Data on CA-related deaths were sourced from annual mortality databases kept by the National Statistics Institute of Spain (1999–2013). Based on the ICD-10, only CAs corresponding to rare diseases definition were included in this study. Annual age-adjusted mortality rates were calculated and time trends were evaluated by joinpoint regression analysis. Geographical differences were assessed using standardised mortality ratios and cluster detection. A total of 13,660 rare-CA-related deaths (53.4% males) were identified in the study period. Annual age-adjusted mortality rates decreased by an average of −5.2% (−5.5% males, −4.8% females, *p* < 0.001). Geographical analysis showed a higher risk of rare-CA-related mortality in regions largely located in the south of the country. Despite their limitations, mortality statistics are essential and useful tools for enhancing knowledge of rare disease epidemiology and, by extension, for designing and targeting public health actions. Monitoring rare-CA-related mortality in Spain has shown a 15-year decline and geographical differences in the risk of death, all of which might well be taken into account by the health authorities in order to ensure equality and equity, and to adopt appropriate preventive measures.

## 1. Introduction

Congenital anomalies (CAs) comprise a large, highly heterogeneous group of birth outcomes, usually classified according to the specific organ or system affected. CAs are an important cause of premature death, chronic illness and lifelong disability worldwide. According to the 2015 Global Burden of Disease study, CAs led to 8.5% (7.7–9.5%) of deaths under the age of 5 years [1]. Liu et al., using vital registration data, also concluded that CAs were the most important cause of death in countries with very low (<10 per 1000 livebirths) and low (10–25 per 1000 livebirths) under-5 mortality rates [2]. It is also clear that CA-related mortality merits attention since, while nearly all leading causes of death registered some form of decrease from 2005 to 2015 [1], CAs, along with neonatal sepsis, were the exception without any significant change. Moreover, Oza et al. observed that the proportion of deaths from congenital disorders was relatively stable across the period (data for 2000–2013 in 194 countries), with the smallest relative decrease in risk being predicted for congenital disorders [3].

As one of the leading causes of infant mortality, monitoring CA-related deaths is a useful practice for epidemiological analysis of population trends, for surveillance, and for research geared to identifying possible risk factors and, by extension, establishing public health actions. Indeed, this is precisely one of the purposes of international initiatives focused on effective birth-defect surveillance, such as the European network of population-based registries for the epidemiologic monitoring of CAs (EUROCAT) or the International Clearinghouse for Birth Defects Surveillance and Research (ICBDSR) [4,5]. These are examples of successful collaborative networks of CA registries and programmes confronting varying degrees of coverage in associated countries [6].

Furthermore, most CAs are low-prevalence conditions and thus individually regarded as rare diseases (prevalence below 5 per 10,000 in the European Union). Indeed, rare CAs account for approximately 80% of subgroups used by EUROCAT for the monitoring of CAs [7]. Although epidemiological studies on rare diseases face different challenges, such as assembling large cohorts of affected individuals, there is continuous encouragement to analyse and enhance the available epidemiological information [8]. In this respect, official nationwide statistics provide uniform population-based data, which are useful as a complement to data from existing surveillance networks and disease-specific or patient registries. More specifically, mortality statistics cover 100% of the population and share some minimum criteria that facilitate temporal and spatial analysis. Accordingly, they furnish uniform, robust series for epidemiological study of low-prevalence diseases [9]. 

The use of Geographic Information Systems (GIS) and spatial analysis data has become commonplace in health research because of the potential for monitoring and tracking disease trends, cluster detection, and/or evaluating environmental hazards [10,11]. Hence, an increasing number of studies have been combining GIS and epidemiological methods, as applied, for instance, to specific rare congenital diseases [12,13] or to CAs as a whole [14,15,16]. Aside from studies published on the mortality of some CAs or small groups of CAs, however, to date there have been no specific temporal or spatial analyses that have focused on the rare CA group as such, and only some items of related useful information can be extracted from other analyses [17].

Accordingly, this study sought to analyse population-based mortality attributed to rare CAs, globally and by anatomic system according to the International Classification of Diseases, as well as for remarkable rare CAs, and assess the associated time trends and geographical differences in Spain. This information can be added to the studies on the occurrence of rare CAs, thus completing the background. It involved obtaining essential knowledge for better characterisation of nationwide distribution of this group of rare diseases, enhancing their visibility, and detecting local neighbourhood clusters which displayed a high risk of death from rare CAs. All this helps in establishing a baseline that can serve as reference for comparisons with future analyses (what can contribute to estimate the impact of possible modifying factors or measures influencing mortality attributed to rare CAs) or with comparable figures in other countries. 

## 2. Materials and Methods

Deaths due to CAs were sourced from annual mortality databases kept by the National Statistics Institute (NSI) of Spain, corresponding to population-based data for the period 1999–2013. Only ICD-10 (International Classification of Diseases, 10th Revision) codes deemed to be rare diseases by reason of their low prevalence were included for study purposes [18]. Therefore, we have considered as *rare CAs* those with a birth prevalence below 5 per 10,000. Causes of death due to rare CAs were grouped by ICD-10 category for the main types of CAs (Table S1), with a breakdown by date and place of death, sex, and date of birth. Annual populations categorised by sex and age at a municipal level were also obtained from the NSI, in order to calculate age-adjusted mortality rates for males, females and both sexes combined (expressed per 100,000 inhabitants). For the age-adjusted mortality rates, we used the Standard European Population as reference.

Time trends were assessed by joinpoint regression analysis, including two possible joinpoints across the 15 years of study. These regression models were calculated overall and by type of CA. The only exceptions were “*cleft lip and cleft palate*”, “*CAs of eye, ear, face and neck*”, and “*CAs of genital organs*”, and this can be explained because although these three groups of rare CAs are included in the present study, their time trend analyses were not performed due to the extremely low number of deceases (6 in total) attributed to these CAs in the period 1999–2013.

Spatial analysis was performed for NSI population and mortality data, by municipality, sex and age group. Municipalities were aggregated into 326 districts, defined as divisions of Spanish territory pertaining to adjacent municipalities having similar geographical and historical features [19]. This spatial unit was chosen for the purposes of robustness and the stability of results based on a low number of deaths [20]. For the period 1999–2013, standardised mortality ratios (SMRs) were calculated by district, and subsequently smoothed in line with the conditional autoregressive model proposed by Besag et al. [21]. The Spanish mortality rate due to rare CAs across the whole period was taken as reference, so it corresponded to the SMR value of 1.00 (expected mortality). Smoothed SMRs make use of data from adjacent units, assuming a Poisson distribution and taking into account the spatial contiguity and heterogeneity of each unit. This enabled us to estimate the relative risk (RR) of death due to rare CAs by district and the associated posterior probability (PP). PP values show those districts with significantly higher (PP > 0.80) or lower (PP < 0.20) risk of death with respect to the expected for the country as reference. The geographical analysis was completed by cluster detection, with a radius of zero to 50 km being set around each of the main municipal population centres. The clusters are assessed as the circles with the maximum likelihood of containing more or fewer cases of rare-CA-related mortality than expected. Results were evaluated using the Monte Carlo simulation (999 iterations) with a 95% confidence interval (CI) [22]. All statistical analyses were performed using the Stata (StataCorp, College Station, TX, USA), Joinpoint (National Cancer Institute, Bethesda, MD, USA), R-INLA (Norwegian University of Science and Technology, Trondheim, Norway) and SaTScan (Martin Kulldorf, Harvard Medical School and Harvard Pilgrim Health Care Institute, Boston, MA, USA) computer software programmes, with ArcGIS software (Esri, Redlands, CA, USA) being used for cartographical representations.

### Research Ethics

The study was conducted in accordance with the Declaration of Helsinki, and the protocol was approved by the Ethics Committee of the Instituto de Salud Carlos III (CEI 50/2013).

## 3. Results

In Spain, there were 13,660 deaths (53.4% males, 46.6% females) due to rare CAs along the period 1999–2013. In terms of type of CA, the highest percentage (40.3%) of deaths corresponded to rare CAs of the circulatory system, followed by a 16.9% due to chromosomal abnormalities (not elsewhere classified), 14.5% due to other congenital malformations, and 9.2% due to rare CAs of the nervous system. In addition to the type of CA, Table 1 shows the distribution of deaths according to EUROCAT subgroups of congenital anomalies, classified by anatomic system. 

Distribution of deaths by age showed that 49.9% occurred in the first year of life, 6.3% occurred between the ages of 1 and 4 years, and the remaining 43.8% occurred later in life. The average age at death was 20.5 years (95% CI: 20.1–21.0), with this being higher among women (21.7 years; 95% CI: 21.0–22.4) than men (19.4 years; 95% CI: 18.9–20.1). From 1999 to 2013, the average age at death increased significantly, rising to nearly double the initial figure by the end of the period, i.e., from 16.6 years (95% CI: 15.1–18.1) in 1999 to 30.3 years (95% CI: 28.0–32.5) in 2013.

### 3.1. Time Trends

**Figure 1:**
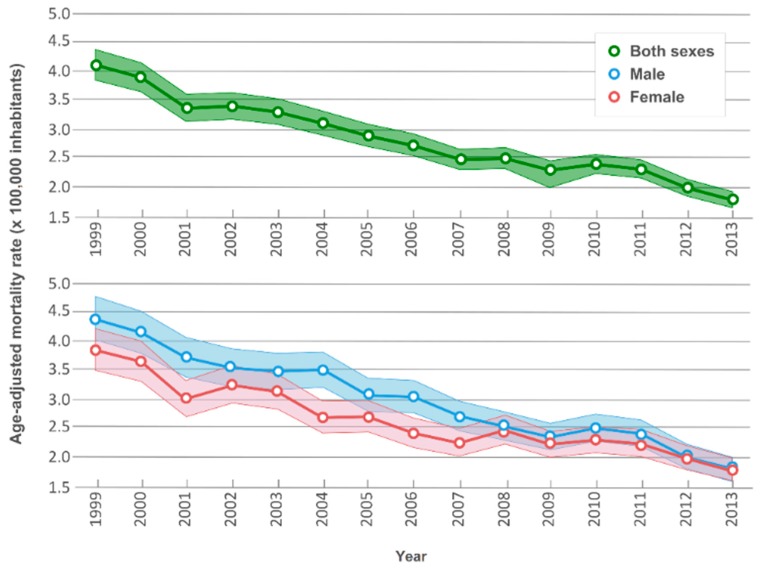
Time trends in age-adjusted mortality rates per 100,000 inhabitants for rare congenital anomalies. Shading represents 95% confidence intervals.

Annual age-adjusted mortality rates displayed downward trends (Figure 1), decreasing from 4.11 (95% CI: 3.86–4.38) per 100,000 inhabitants in 1999 to 1.77 (95% CI: 1.63–1.91) per 100,000 inhabitants in 2013 (annual percentage change, APC: −5.2%, *p* < 0.001). This downward trend was reflected in both sexes, without any joinpoint, with a fall in male age-adjusted mortality rates from 4.39 (95% CI: 4.03–4.78) in 1999 to 1.79 (95% CI: 1.60–1.99) in 2013 (APC: −5.5%, *p* < 0.001), and in female rates from 3.84 (95% CI: 3.50–4.22) in 1999 to 1.76 (95% CI: 1.57–1.97) in 2013 (APC: −4.8%, *p* < 0.001). 

Table 2 includes the annual age-adjusted mortality rates (ARs) by sex and type of rare CA. Apart from the data for the CA grouped by body systems, it also includes defect specific results for all the defects for which at least 180 deaths (i.e., at least an average of 12 deaths per year) were observed in the study period. According to data shown by type of rare CA, the greatest fall corresponded to transposition of the great vessels, followed by hydrocephalus, hypoplastic left heart, and severe CHD as a group. Time trends were not significant for rare CAs of the digestive system, as well as neural tube defects, and chromosomal abnormalities (not elsewhere classified) and trisomy 18 in males, and rare CAs of the respiratory system and urinary system in women (Table 2).

### 3.2. Geographical Distribution

Table 3 shows the districts in which SMRs differed from the expected value for Spain along the 15 years studied (SMR = 1.00). As can be seen from the values registered for both sexes, lower-than-expected mortality was detected in 14 districts situated in the provinces of Alicante, Badajoz, Balearic Islands, Barcelona, Girona, Madrid, Pontevedra, Cantabria and Toledo. SMRs were higher than expected in 24 districts: of these, 20 (83.3%) corresponded to provinces lying in the south of Spain (Almería, Cádiz, Córdoba, Granada, Jaén, Málaga, Las Palmas, Tenerife, Seville, Ceuta and Murcia), two corresponded to provinces lying in the north (Asturias and León), and two corresponded to provinces lying in the west (Cáceres and Badajoz). 

**Figure 2:**
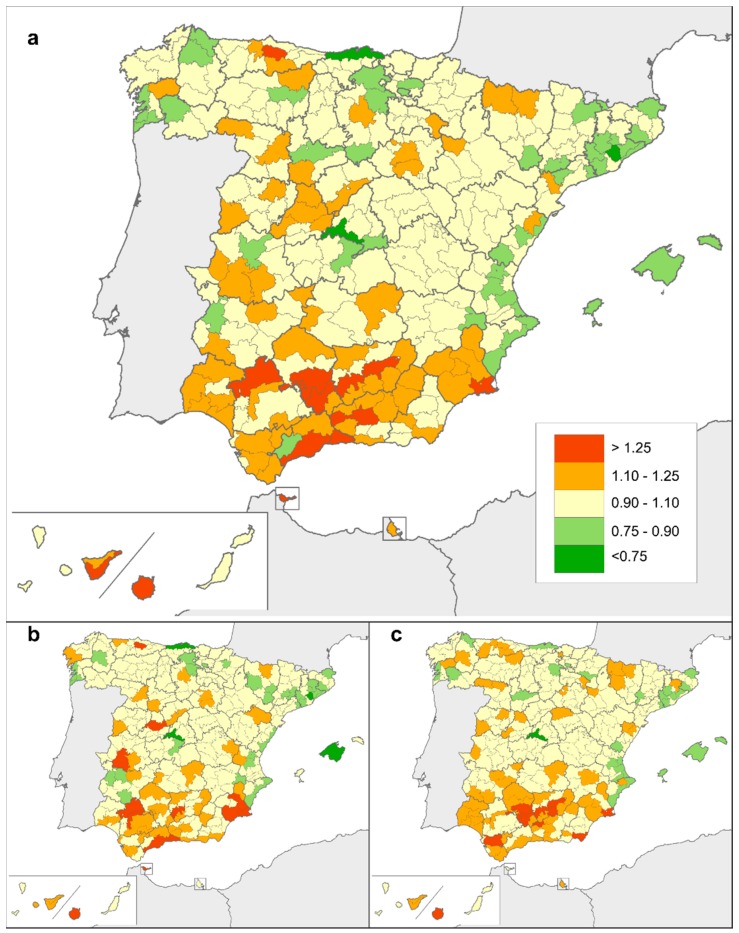
Smoothed standardised mortality ratios (smoothed SMRs) for rare congenital anomalies: (**a**) both sexes, (**b**) males, (**c**) females.

**Figure 3:**
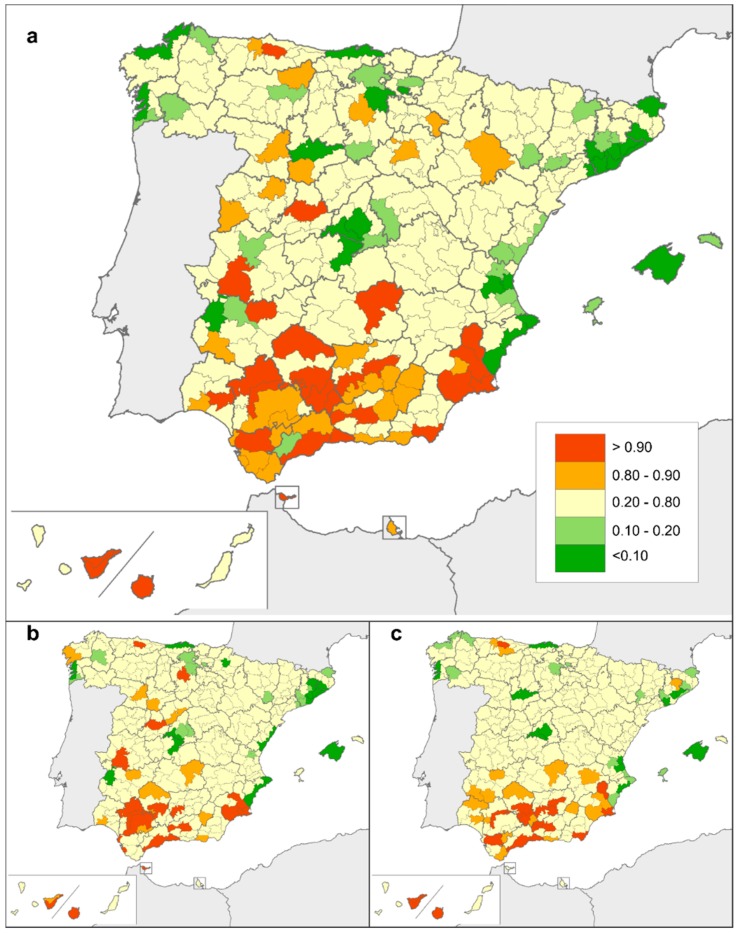
Posterior probability (PP) values for: (**a**) both sexes, (**b**) males, (**c**) females. PP shows those districts with significantly higher (PP > 0.80) and lower (PP < 0.20) than expected (for the country as reference) risk of death due to rare congenital anomalies.

Detailed mapping made it easier to monitor spatial differences in risk of death due to rare CAs. Figure 2 depicts the geographical variability in smoothed SMRs, taking into account mortality registered in each district and its adjacent districts. According to the PP values associated with these smoothed SMRs, risk of death due to rare CAs was significantly higher than expected in districts situated in the south of Spain, with some exceptions (Figure 3). This geographical pattern remained unchanged when males and females were analysed separately. 

**Figure 4:**
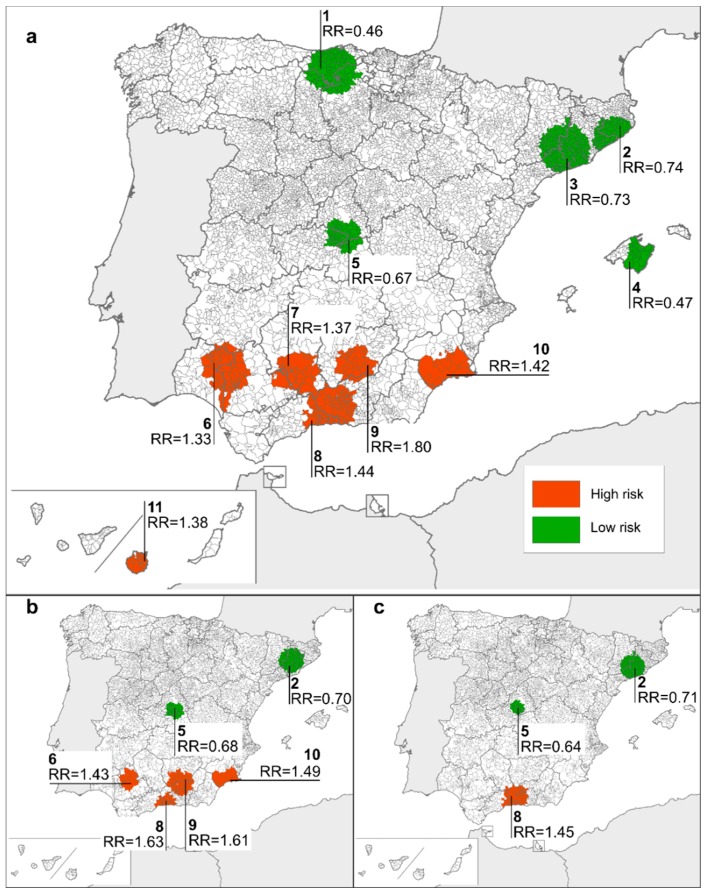
Rare congenital-anomaly mortality clusters for: (**a**) both sexes, (**b**) males, (**c**) females. Clusters are named according to Spanish provinces involved: 1 = Cantabria, Burgos, Palencia; 2 = Girona, Barcelona; 3 = Barcelona, Tarragona, Lleida; 4 = Mallorca; 5 = Madrid, Toledo; 6 = Huelva, Seville; 7 = Córdoba, Seville; 8 = Granada, Málaga; 9 = Jaén; 10 = Murcia; 11 = Gran Canaria.

Lastly, the above mapping exercise was completed by identification of spatial clustering, with a higher-than-expected risk of mortality being detected in 6 clusters of municipalities situated in the south, and a lower risk being identified in the north (3 clusters), east (1 cluster) and centre (1 cluster) of the country. As before, the geographical distribution of clusters from both sexes combined, remained unchanged when males and females were analysed separately, and less significant groups were identified (Figure 4).

## 4. Discussion

Mortality is a major health-status indicator which is fairly well monitored for some common diseases [23]. Unfortunately, there is still a sizeable knowledge gap for large groups of rare diseases, and too many aspects relating to rare-disease mortality remain unknown. Rare CAs are not an exception, and not many studies have addressed the associated mortality. Such research is essential because, while mortality remains unknown, any interpretation of lifetime prevalence could well prove misleading. Failure to consider mortality data could lead to the conclusion that a disease was negligible if it caused death very early in life. This, in turn, could prevent the allocation of the precise health resources that could increase survival or survival under better conditions.

This population-based study on rare CAs shows the continuous downward trend in CA-related mortality and the geographical distribution of risk of death from these causes in Spain. In addition, it confirms that most deaths occur below 5 years of age, with the first year of life accounting for half of all CA-related deaths. Moreover, the average age at death caused by rare CAs corresponds to early adulthood (around 20 years old). Although such average age has significantly increased along the time (in accordance to the downward trend in CA-related mortality), it seems clear that greater efforts are needed to ascertain the exact determinants of such early death, in order to establish the most appropriate prevention measures. Nevertheless, this scenario is not stable. In a matter of just 15 years, the average age of CA-related death has doubled, which is a promising sign. This goes to show that, insofar as CAs are concerned, a change is taking place in the Spanish population. Moreover, given the degree of geographical heterogeneity that was detected, the determinants of CA-related mortality may not be uniformly distributed across the country, and special attention should therefore be paid to areas with the highest rates, and even to those with the lowest rates because, to some extent, such areas might serve as models. Comparison of these two types of areas might conceivably yield some clues for prevention.

One of our study’s most relevant findings is the above mentioned observed fall in age-adjusted mortality rates from 1999 to 2013 across all the different subgroups of rare CAs (with the exception of those of the digestive system, for which no statistically significant decrease was found). This decrease (for which we ruled out methodological issues as a cause) is in line with the increase in the average age at death attributed to rare CAs, since both are interrelated. Both findings could also be due to a number of other reasons. The treatment and care of patients with CAs—even prenatally—likely had an influence on the mortality figures, though lack of data means that this cannot be quantified. Similarly, advances in prenatal diagnosis make for better preparedness and the referral of the deliveries of severely affected pregnancies to tertiary hospitals, where more adequate care can be provided at birth and during the neonatal period. It has been shown, for instance, that prenatal diagnosis of congenital heart defects allows for early preemptive stabilisation, and is associated with improved early clinical status [24]. Improved prenatal diagnosis also must have an impact, in the sense that better detection of CAs in the foetus increases the likelihood of interruption of affected pregnancies. This means that a higher number of elective terminations of pregnancy due to foetal anomalies (ETOPFA) will reduce the number of affected newborn infants, and this may in turn affect the mortality figures. In fact, infant CA mortality in a given country is higher when prevalence of ETOPFA is lower, and it thus follows that increases over time in the ETOPFA rate would tend to lower the infant mortality rate [25]. Furthermore, the severity of the defects has to be considered, in that the most severely affected cases (those with a higher risk of postnatal death) will more probably be detected prenatally, with a considerable number of subsequent ETOPFA, thereby also influencing mortality figures by reducing them.

It should be said that, based on EUROCAT data, the prevalence of some CAs, particularly severe congenital heart defects, was reported to have increased in Europe, from 2004 to 2012 [25]. While these data also included ETOPFA, the number or proportion of ETOPFA among the cases was not specified. Hence, the increase *per se* could not be taken to mean that the number of newborn infants with these types of defects also increased. The authors speculated that this might reflect increases in maternal obesity and diabetes, both of which are well-known risk factors for CAs. For the purposes of our study, if the number of ETOPFA was high, this could in fact have reduced the postnatal mortality figure. This is a good example of the complexity of the situation and its interpretation.

It is noteworthy that 56.1% of deaths attributed to rare CAs occur before the age of 5 years, and even more striking that 49.9% occur in the first year of life. This is an important item of information because it narrows down the age at which the risk of death is highest, and consequently the age at which follow-up should somehow be different, so as to ensure that the determinants of early death are properly approached. Furthermore, it provides some evidence of the need for strategic allocation of resources specifically required for that segment of the population. This facilitates analysis and identification of the hospital(s) and local or district services that should be reinforced, through strengthening the workforce and/or providing adequate materials and infrastructure. Another strategy for reducing CA-related mortality could lie in designating some national reference hospitals, services and units specialised in the care of specific diseases (here in Spain the equivalent designation is known as Reference Centres, Services, and Units of the National Health System; *Centros, Servicios y Unidades de Referencia*, CSUR). There are data in the literature showing, for instance, that the lower number of patients attended at heart surgery centres is associated with higher neonatal mortality among cases with transposition of the great arteries [26].

In terms of geographical distribution, risk of death was observed to be consistently higher in the south of Spain, and specifically in certain districts. While this type of epidemiological finding can sometimes generate concern among the population [11], it should preferably be seen as indicating where in-depth research would be needed into the causes of that distribution of mortality. Further analyses, beyond the scope of this study, could reveal some possible ways of minimising the risk.

Our findings could prove useful as a base for comparison with other countries, or as a reference in time to assess possible changes and the impact of different measures in the future. In contrast to other approaches that use multisource data-integration for rare-CA prevalence estimates [7], we propose that the results of this nationwide registry-based study on mortality be used as a potential contributor for the monitoring of rare CAs. This could be a simple, effective, complementary way of improving epidemiological surveillance in countries addressing current difficulties in pooling cases from different registries (i.e., individual linkage between CAs and rare–disease registries) although of course, mortality statistics should not be considered an optimal source of data for case identification. It could also benefit those programmes that form part of the EUROCAT network but have incomplete geographical coverage of their country, such as the population-based CA registries in Spain [4].

Regarding the clinical impact of CAs, it is evident the influence of the important cost associated with birth defects [27], as well as the increase in disability adjusted life years (DALYs) rates and years lived with disability (YLDs) for CAs [1,28]. In addition, terminations of pregnancy for CAs were almost three times more frequent than the combination of infant deaths and stillbirths with CA, which clearly must affect the global burden of disease [17], and this should be taken into account when interpreting any figure.

This retrospective descriptive study has several limitations, such as the inability to link exposure to outcome in individuals, and to control for confounding factors. Therefore, it cannot be used to determine an association between a risk factor and disease. Consequently, additional research in this sense is needed for rare CAs.

Although the use of underlying cause of death underestimates case identification when compared to multiple-cause analysis, it is nonetheless an effective approach to mortality directly attributable to rare CAs. Some authors have estimated that mortality due to congenital anomalies for the under-5 age group is likely to be a fourfold underestimate [29]. If this were also applied to Spain, it would mean that the situation could be rather striking, something that yet again would make it advisable to focus attention on this younger stratum of the population. On the other hand, differences in diagnostic quality or coding practices over time or among regions might bias our results, even though the death registry officially follows a standardised, uniform methodology.

The use of ICD for rare diseases research is challenging and in fact the lack of appropriate coding makes difficult, and sometimes impossible, the study of a particular CA without a specific ICD code [8]. In this paper, the analysis of rare CAs (globally and by system) provides a general view of this public health problem, even though some misclassification issues cannot completely be ruled out.

Considering quality issues of deaths certificates, the differences and incompatibilities between original underlying cause of death and final main condition were assessed previously [30]. In that study, Johansson and Westerling reported the lowest percentage of differences for CAs, in comparison to other ICD chapters, which is reassuring.

Despite these limitations mortality statistics provide broad temporal and geographical coverage and continue to be a very useful tool for studying the epidemiology of low–prevalence diseases [9,20], either for epidemiological research as well as for health monitoring [30]. In addition, mortality databases enhance the ability to collect cases diagnosed after the early neonatal period (lifetime detection), thereby becoming a complementary data source for rare-CA studies.

## 5. Conclusions

In conclusion, this is the first nationwide population-based study to focus on mortality due to rare CAs. Our results contribute to the monitoring of rare CAs along 15 years in Spain, by providing evidence of the continuous decline in mortality rates and illustrate some geographical differences in the risk of death. These findings are not only useful for assessing the burden of low-prevalence CAs in Spain, but also serve as evidence which might be taken into account by health authorities, in order to identify possible risk factors, adopt appropriate preventive measures, implement and evaluate health policies and healthcare plans ensuring equality and equity, with the ultimate purpose of achieving better health for all.

## Figures and Tables

**Table 1:** Number of deaths registered in Spain (1999–2013) by underlying cause of death, this being a rare CA. The following list of rare congenital anomalies is based on EUROCAT subgroups of congenital anomalies [4]. Only those EUROCAT defects considered as rare diseases with corresponding ICD-10 codes are displayed.

Rare CAs as Cause of Death		Number of Deaths		
		Total	Men	Women
**Rare CAs of the Nervous System**				
**Nervous system**	**Q00, Q01, Q02, Q03, Q04, Q05, Q06, Q07**	**1251**	**645**	**606**
Neural Tube Defects	Q00, Q01, Q05	272	140	132
Anencephalus and similar	Q00	97	49	48
Encephalocele	Q01	15	10	5
Myelomeningocele/Spina Bifida	Q05	160	81	79
Hydrocephalus	Q03	199	119	80
Severe microcephaly	Q02	54	24	30
Arhinencephaly/holoprosencephaly	Q04.1, Q04.2	59	24	35
**Rare CAs of the Eye, Ear, Face and Neck**				
**Eye**	**Q10–Q15**	**1**	**0**	**1**
Anophthalmos/microphthalmos	Q11.0, Q11.1, Q11.2	1	0	1
Anophthalmos	Q11.0, Q11.1	0	0	0
Congenital cataract	Q12.0	0	0	0
Congenital glaucoma	Q15.0	0	0	0
**Ear, face and neck**	**Q16, Q17, Q18**	**0**	**0**	**0**
Anotia	Q16.0	0	0	0
**Rare CAs of the Circulatory System**				
**Congenital Heart Defects (CHD)**	**Q20–Q26**	**4927**	**2780**	**2147**
Severe CHD	Q20.0, Q20.1, Q20.3, Q20.4, Q21.2, Q21.3, Q22.0, Q22.4, Q22.5, Q22.6, Q23.0, Q23.2, Q23.3, Q23.4, Q25.1, Q25.2, Q26.2	1475	841	634
Common arterial truncus	Q20.0	44	20	24
Double outlet right ventricle	Q20.1	19	12	7
Transposition of great vessels	Q20.3	235	151	84
Single ventricle	Q20.4	75	40	35
AVSD	Q21.2	126	52	74
Tetralogy of Fallot	Q21.3	360	210	150
Triscuspid atresia and stenosis	Q22.4	34	15	19
Ebstein’s anomaly	Q22.5	75	32	43
Pulmonary valve stenosis	Q22.1	6	4	2
Pulmonary valve atresia	Q22.0	5	2	3
Aortic valve atresia/stenosis	Q23.0	57	37	20
Mitral valve anomalies	Q23.2, Q23.3	27	15	12
Hypoplastic left heart	Q23.4	256	155	101
Hypoplastic right heart	Q22.6	5	3	2
Coarctation of aorta	Q25.1	150	93	57
Total anomalous pulmonary venous return	Q26.2	7	4	3
PDA as only CHD in term infants (GA + 37 weeks)	Q25.0	290	155	135
**Rare CAs of the Respiratory System**				
**Respiratory**	**Q30.0, Q32–Q34**	**347**	**203**	**144**
Choanal atresia	Q30.0	8	5	3
**Cleft lip and Cleft Palate**				
**Oro-facial clefts**	**Q35–Q37**	**4**	**1**	**3**
Cleft lip with or without cleft palate	Q36, Q37	0	0	0
Cleft palate	Q35	4	1	3
**Rare CAs of the Digestive System**				
**Digestive system**	**Q38–Q45**	**1092**	**593**	**499**
Oesophageal atresia with or without tracheaoesophageal fistula	Q39.0–Q39.1	107	55	52
Duodenal atresia or stenosis	Q41.0	11	7	4
Atresia or stenosis of other parts of small intestine	Q41.1–Q41.8	17	9	8
Ano-rectal atresia and stenosis	Q42.0–Q42.3	6	4	2
Hirschsprung’s disease	Q43.1	47	27	20
Atresia of bile ducts	Q44.2	55	30	25
Annular pancreas	Q45.1	0	0	0
**Rare CAs of the Urinary System**				
**Urinary**	**Q60–Q64, Q79.4**	**756**	**393**	**363**
Bilateral renal agenesis including Potter syndrome	Q60.1, Q60.6	84	53	31
Renal dysplasia	Q61.4	28	20	8
Bladder exstrophy and/or epispadia	Q64.0, Q64.1	2	2	0
Posterior urethral valve and/or prune belly	Q64.2, Q64.3, Q79.4	7	6	1
**Rare CAs of Genital Organs**				
**Genital**	**Q50–Q52, Q54–Q56**	**1**	**1**	**0**
Hypospadias	Q54	1	1	0
Indeterminate sex	Q56	0	0	0
**Rare CAs of the Musculoskeletal System**				
Craniosynostosis	Q75.0	6	3	3
Diaphragmatic hernia	Q79.0	339	191	148
**Limb**	**Q65–Q74**	**56**	**24**	**32**
Limb reduction defects	Q71–Q73	0	0	0
Polydactyly	Q69	0	0	0
Syndactyly	Q70	0	0	0
**Abdominal wall defects**	**Q79.2, Q79.3, Q79.5**	**66**	**42**	**24**
Gastroschisis	Q79.3	44	30	14
Omphalocele	Q79.2	22	12	10
**Other Rare Congenital Malformations**				
**Other anomalies/syndromes**				
Congenital skin disorders	Q80–Q82	93	62	31
Fetal alcohol syndrome	Q86.0	2	2	0
Situs inversus	Q89.3	31	16	15
Conjoined twins	Q89.4	11	2	9
**Rare Chromosomal Abnormalities, not elsewhere classified**				
**Chromosomal**	**Q90–Q92, Q93, Q96–Q99**	**2312**	**1068**	**1244**
Patau syndrome/trisomy 13	Q91.4–Q91.7	143	62	81
Edwards syndrome / trisomy 18	Q91.0–Q91.3	288	81	207
Turner syndrome	Q96	23	1	22
Klinefelter syndrome	Q98.0–Q98.4	3	3	0

**Table 2:** Annual age-adjusted mortality rates (ARs) and 95% confidence intervals (CIs), by sex and type of rare congenital anomaly. Time trends results are shown as annual percentage change (APC) and *p*-value.

**Year**	**Rare CAs of the Nervous System**						**Neural Tube Defects**					
	**Both Sexes**		**Men**		**Women**		**Both Sexes**		**Men**		**Women**	
	***n***	**AR (CI)**	***n***	**AR (CI)**	***n***	**AR (CI)**	***n***	**AR (CI)**	***n***	**AR (CI)**	***n***	**AR (CI)**
1999	93	0.37 (0.29–0.45)	47	0.35 (0.25–0.47)	46	0.39 (0.28–0.53)	23	0.10 (0.06–0.15)	11	0.08 (0.04–0.15)	12	0.12 (0.06–0.20)
2000	95	0.36 (0.29–0.45)	51	0.37 (0.27–0.50)	44	0.35 (0.25–0.48)	25	0.10 (0.06–0.15)	12	0.09 (0.05–0.16)	13	0.10 (0.05–0.18)
2001	87	0.34 (0.27–0.43)	54	0.42 (0.32–0.56)	33	0.26 (0.17–0.37)	24	0.09 (0.06–0.14)	15	0.11 (0.06–0.19)	9	0.07 (0.03–0.14)
2002	79	0.29 (0.23–0.37)	34	0.25 (0.17–0.35)	45	0.35 (0.25–0.47)	15	0.07 (0.04–0.11)	9	0.08 (0.04–0.15)	6	0.06 (0.02–0.12)
2003	93	0.34 (0.27–0.42)	47	0.34 (0.25–0.46)	46	0.34 (0.25–0.46)	20	0.07 (0.04–0.11)	14	0.10 (0.05–0.17)	6	0.05 (0.02–0.10)
2004	95	0.31 (0.32–0.39)	56	0.38 (0.28–0.50)	39	0.26 (0.18–0.36)	18	0.06 (0.03–0.10)	9	0.05 (0.02–0.11)	9	0.06 (0.03–0.13)
2005	82	0.26 (0.21–0.33)	45	0.29 (0.21–0.40)	37	0.24 (0.16–0.33)	16	0.05 (0.03–0.09)	10	0.07 (0.03–0.13)	6	0.03 (0.01–0.08)
2006	80	0.25 (0.21–0.33)	46	0.28 (0.20–0.37)	34	0.23 (0.16–0.32)	21	0.06 (0.04–0.10)	12	0.06 (0.03–0.12)	9	0.06 (0.03–0.11)
2007	80	0.25 (0.20–0.31)	41	0.25 (0.18–0.34)	39	0.25 (0.18–0.35)	16	0.05 (0.03–0.08)	6	0.04 (0.01–0.09)	10	0.06 (0.03–0.11)
2008	73	0.22 (0.18–0.28)	35	0.21 (0.14–0.29)	38	0.25 (0.17–0.34)	16	0.05 (0.03–0.08)	11	0.07 (0.03–0.12)	5	0.03 (0.01–0.08)
2009	74	0.21 (0.17–0.27)	33	0.19 (0.13–0.26)	41	0.24 (0.17–0.34)	20	0.06 (0.03–0.09)	8	0.05 (0.02–0.10)	12	0.07 (0.03–0.12)
2010	93	0.26 (0.21–0.32)	42	0.24 (0.17–0.32)	51	0.28 (0.21–0.38)	12	0.03 (0.01–0.05)	4	0.02 (0.01–0.06)	8	0.04 (0.01–0.08)
2011	82	0.22 (0.17–0.27)	44	0.23 (0.16–0.31)	38	0.20 (0.14–0.29)	18	0.04 (0.02–0.07)	6	0.03 (0.01–0.06)	12	0.06 (0.03–0.10)
2012	77	0.21 (0.16–0.26)	37	0.21 (0.14–0.29)	40	0.20 (0.14–0.28)	14	0.03 (0.02–0.05)	6	0.03 (0.01–0.06)	8	0.04 (0.01–0.08)
2013	68	0.20 (0.15–0.25)	33	0.18 (0.12–0.26)	35	0.22 (0.15–0.31)	14	0.04 (0.02–0.06)	7	0.04 (0.01–0.08)	7	0.04 (0.01–0.08)
APC	−4.49 (*p* < 0.001)		−5.19 (*p* < 0.001)		−3.87 (*p* < 0.001)		−6.60 (*p* < 0.001)		−2.40 (*p* = 0.30)		−8.24 (*p* < 0.001)	
**Year**	**Hydrocephalus**						**Rare CAs of the Circulatory System**					
	**Both Sexes**		**Men**		**Women**		**Both Sexes**		**Men**		**Women**	
	***n***	**AR (CI)**	***n***	**AR (CI)**	***n***	**AR (CI)**	***n***	**AR (CI)**	***n***	**AR (CI)**	***n***	**AR (CI)**
1999	20	0.08 (0.05–0.13)	14	0.11 (0.06–0.19)	6	0.05 (0.02–0.11)	486	1.90 (1.73–2.09)	274	2.11 (1.86–2.38)	212	1.69 (1.46–1.95)
2000	22	0.09 (0.05–0.14)	13	0.10 (0.05–0.18)	9	0.07 (0.03–0.14)	480	1.85 (1.68–2.03)	268	2.06 (1.82–2.34)	212	1.63 (1.40–1.88)
2001	12	0.05 (0.02–0.08)	6	0.04 (0.02–0.10)	6	0.05 (0.02–0.11)	387	1.51 (1.36–1.67)	224	1.69 (1.46–1.93)	163	1.33 (1.12–1.56)
2002	22	0.08 (0.05–0.12)	10	0.07 (0.03–0.13)	12	0.09 (0.05–0.17)	421	1.60 (1.44–1.76)	241	1.79 (1.57–2.04)	180	1.39 (1.19–1.62)
2003	15	0.05 (0.03–0.09)	7	0.05 (0.02–0.10)	8	0.06 (0.02–0.12)	391	1.38 (1.24–1.53)	211	1.48 (1.28–1.70)	180	1.28 (1.09–1.49)
2004	16	0.05 (0.03–0.09)	12	0.08 (0.04–0.14)	4	0.03 (0.01–0.07)	404	1.39 (1.25–1.53)	254	1.71 (1.50–1.94)	150	1.05 (0.88–1.24)
2005	12	0.04 (0.02–0.07)	10	0.06 (0.03–0.11)	2	0.02 (0.00–0.06)	341	1.16 (1.03–1.29)	205	1.37 (1.19–1.58)	136	0.93 (0.78–1.11)
2006	9	0.03 (0.01–0.06)	7	0.05 (0.02–0.10)	2	0.01 (0.00–0.05)	380	1.21 (1.09–1.34)	220	1.38 (1.20–1.58)	160	1.04 (0.88–1.22)
2007	11	0.03 (0.02–0.06)	7	0.04 (0.02–0.09)	4	0.03 (0.01–0.08)	318	0.99 (0.88–1.11)	170	1.09 (0.93–1.26)	140	0.9 (0.75–1.07)
2008	14	0.04 (0.02–0.07)	7	0.04 (0.01–0.08)	7	0.05 (0.02–0.10)	350	1.05 (0.94–1.17)	190	1.14 (0.98–1.32)	160	0.95 (0.81–1.12)
2009	9	0.02 (0.01–0.05)	4	0.02 (0.01–0.06)	5	0.03 (0.01–0.07)	341	0.97 (0.86–1.08)	190	1.06 (0.91–1.23)	151	0.87 (0.73–1.02)
2010	13	0.04 (0.02–0.06)	8	0.05 (0.02–0.09)	5	0.02 (0.01–0.06)	349	0.97 (0.87–1.08)	190	1.06 (0.91–1.23)	159	0.88 (0.74–1.03)
2011	10	0.03 (0.01–0.05)	9	0.04 (0.02–0.09)	1	0.01 (0.00–0.04)	321	0.9 (0.80–1.01)	184	1.01 (0.87–1.17)	137	0.8 (0.66–0.95)
2012	6	0.02 (0.01–0.03)	1	0.01 (0.00–0.03)	5	0.02 (0.01–0.06)	295	0.81 (0.71–0.91)	153	0.84 (0.71–0.99)	142	0.77 (0.64–0.92)
2013	8	0.02 (0.01–0.05)	4	0.02 (0.01–0.06)	4	0.02 (0.01–0.06)	246	0.67 (0.59–0.77)	134	0.71 (0.59–0.85)	112	0.64 (0.52–0.78)
APC	−9.65 (*p* < 0.001)		−9.11 (*p* < 0.001)		−9.08 (*p* < 0.001)		−6.44 (*p* < 0.001)		−6.70 (*p* < 0.001)		−6.08 (*p* < 0.001)	
**Year**	**Severe CHD**						**Transposition of Great Vessels**					
	**Both Sexes**		**Men**		**Women**		**Both Sexes**		**Men**		**Women**	
	***n***	**AR (CI)**	***n***	**AR (CI)**	***n***	**AR (CI)**	***n***	**AR (CI)**	***n***	**AR (CI)**	***n***	**AR (CI)**
1999	145	0.59 (0.50–0.70)	89	0.72 (0.57–0.89)	56	0.47 (0.35–0.62)	28	0.12 (0.08–0.18)	18	0.16 (0.09–0.25)	10	0.09 (0.04–0.16)
2000	148	0.59 (0.49–0.69)	75	0.59 (0.46–0.74)	73	0.58 (0.45–0.74)	30	0.13 (0.08–0.18)	18	0.15 (0.09–0.24)	12	0.10 (0.05–0.18)
2001	100	0.39 (0.32–0.48)	56	0.42 (0.31–0.55)	44	0.36 (0.26–0.49)	15	0.06 (0.03–0.10)	9	0.07 (0.03–0.14)	6	0.05 (0.02–0.11)
2002	119	0.46 (0.38–0.56)	66	0.49 (0.38–0.63)	53	0.43 (0.32–0.57)	22	0.09 (0.06–0.14)	15	0.11 (0.06–0.19)	7	0.06 (0.03–0.13)
2003	102	0.38 (0.31–0.47)	52	0.39 (0.29–0.51)	50	0.38 (0.28–0.50)	13	0.05 (0.03–0.09)	7	0.06 (0.02–0.12)	6	0.05 (0.02–0.11)
2004	117	0.40 (0.33–0.49)	79	0.53 (0.42–0.67)	38	0.27 (0.19–0.37)	22	0.08 (0.05–0.12)	13	0.09 (0.05–0.16)	9	0.07 (0.03–0.13)
2005	101	0.33 (0.27–0.41)	58	0.37 (0.28–0.49)	43	0.29 (0.21–0.39)	17	0.06 (0.03–0.09)	13	0.08 (0.04–0.15)	4	0.03 (0.01–0.07)
2006	109	0.35 (0.29–0.43)	60	0.38 (0.29–0.49)	49	0.33 (0.24–0.44)	15	0.05 (0.03–0.08)	9	0.06 (0.02–0.11)	6	0.04 (0.01–0.09)
2007	101	0.31 (0.25–0.38)	63	0.39 (0.29–0.50)	38	0.23 (0.16–0.32)	12	0.04 (0.02–0.07)	9	0.06 (0.03–0.11)	3	0.02 (0.00–0.06)
2008	83	0.26 (0.20–0.32)	48	0.29 (0.21–0.38)	35	0.22 (0.15–0.31)	14	0.05 (0.02–0.08)	7	0.05 (0.02–0.09)	7	0.05 (0.02–0.10)
2009	80	0.23 (0.18–0.29)	51	0.29 (0.21–0.38)	29	0.17 (0.11–0.25)	12	0.04 (0.02–0.07)	9	0.06 (0.03–0.11)	3	0.02 (0.00–0.06)
2010	71	0.19 (0.15–0.25)	38	0.21 (0.14–0.28)	33	0.18 (0.13–0.26)	9	0.03 (0.01–0.05)	6	0.03 (0.01–0.07)	3	0.02 (0.00–0.06)
2011	65	0.18 (0.14–0.23)	35	0.19 (0.13–0.26)	30	0.17 (0.12–0.25)	11	0.03 (0.01–0.05)	9	0.05 (0.02–0.09)	2	0.01 (0.00–0.04)
2012	68	0.19 (0.14–0.24)	36	0.20 (0.14–0.27)	32	0.18 (0.12–0.25)	9	0.03 (0.01–0.05)	6	0.03 (0.01–0.08)	3	0.02 (0.00–0.05)
2013	66	0.18 (0.14–0.24)	35	0.19 (0.13–0.26)	31	0.18 (0.12–0.26)	6	0.02 (0.01–0.04)	3	0.02 (0.00–0.05)	3	0.02 (0.00–0.05)
APC	−8.55 (*p* < 0.001)		−8.59 (*p* < 0.001)		−8.53 (*p* < 0.001)		−11.80 (*p* < 0.001)		−11.03 (*p* < 0.001)		−12.43 (*p* < 0.001)	
**Year**	**Hypoplastic Left Heart**						**Rare CAs of the Respiratory System**					
	**Both Sexes**		**Men**		**Women**		**Both Sexes**		**Men**		**Women**	
	***n***	**AR (CI)**	***n***	**AR (CI)**	***n***	**AR (CI)**	***n***	**AR (CI)**	***n***	**AR (CI)**	***n***	**AR (CI)**
1999	23	0.11 (0.07–0.16)	13	0.12 (0.06–0.21)	10	0.10 (0.05–0.18)	25	0.11 (0.07–0.17)	11	0.10 (0.05–0.18)	14	0.13 (0.07–0.21)
2000	19	0.09 (0.05–0.14)	9	0.08 (0.04–0.16)	10	0.10 (0.05–0.18)	23	0.10 (0.06–0.15)	14	0.12 (0.07–0.21)	9	0.07 (0.03–0.14)
2001	22	0.10 (0.06–0.15)	11	0.10 (0.05–0.18)	11	0.11 (0.05–0.19)	24	0.11 (0.07–0.16)	16	0.14 (0.08–0.22)	8	0.08 (0.03–0.15)
2002	25	0.11 (0.07–0.16)	14	0.12 (0.07–0.20)	11	0.10 (0.05–0.18)	22	0.09 (0.05–0.14)	14	0.11 (0.06–0.19)	8	0.07 (0.03–0.14)
2003	21	0.09 (0.05–0.14)	13	0.11 (0.06–0.18)	8	0.07 (0.03–0.14)	35	0.14 (0.10–0.19)	24	0.18 (0.12–0.28)	11	0.09 (0.04–0.16)
2004	21	0.08 (0.05–0.13)	15	0.12 (0.07–0.19)	6	0.05 (0.02–0.11)	31	0.12 (0.08–0.17)	17	0.12 (0.07–0.20)	14	0.12 (0.06–0.19)
2005	17	0.06 (0.04–0.10)	9	0.07 (0.03–0.13)	8	0.06 (0.03–0.12)	25	0.09 (0.06–0.14)	15	0.11 (0.06–0.18)	10	0.08 (0.04–0.14)
2006	21	0.08 (0.05–0.12)	15	0.10 (0.06–0.17)	6	0.05 (0.02–0.10)	20	0.07 (0.04–0.11)	12	0.08 (0.04–0.14)	8	0.06 (0.02–0.11)
2007	17	0.06 (0.04–0.10)	13	0.09 (0.05–0.15)	4	0.03 (0.01–0.08)	28	0.10 (0.06–0.14)	17	0.11 (0.06–0.18)	11	0.08 (0.04–0.15)
2008	16	0.05 (0.03–0.09)	11	0.07 (0.04–0.13)	5	0.04 (0.01–0.08)	20	0.06 (0.04–0.10)	10	0.07 (0.03–0.12)	10	0.06 (0.03–0.11)
2009	11	0.04 (0.02–0.06)	7	0.04 (0.02–0.09)	4	0.03 (0.01–0.07)	22	0.06 (0.04–0.10)	14	0.08 (0.04–0.14)	8	0.04 (0.02–0.09)
2010	12	0.04 (0.02–0.07)	8	0.05 (0.02–0.10)	4	0.03 (0.01–0.07)	22	0.07 (0.04–0.10)	15	0.09 (0.05–0.14)	7	0.04 (0.02–0.09)
2011	12	0.04 (0.02–0.07)	6	0.04 (0.01–0.08)	6	0.04 (0.01–0.09)	32	0.10 (0.07–0.14)	16	0.10 (0.05–0.16)	16	0.1 (0.06–0.17)
2012	10	0.03 (0.02–0.06)	4	0.02 (0.01–0.06)	6	0.04 (0.01–0.09)	23	0.07 (0.05–0.11)	14	0.08 (0.05–0.14)	9	0.06 (0.03–0.11)
2013	9	0.03 (0.01–0.06)	7	0.04 (0.02–0.09)	2	0.01 (0.00–0.05)	16	0.05 (0.03–0.08)	9	0.06 (0.03–0.11)	7	0.05 (0.02–0.09)
APC	−9.00 (*p* < 0.001)		−7.47 (*p* < 0.001)		−10.53 (*p* < 0.001)		−4.07 (*p* < 0.001)		−4.46 (*p* < 0.001)		−3.72 (*p* = 0.87)	
**Year**	**Rare CAs of the Digestive System**						**Rare CAs of the Urinary System**					
	**Both Sexes**		**Men**		**Women**		**Both Sexes**		**Men**		**Women**	
	***n***	**AR (CI)**	***n***	**AR (CI)**	***n***	**AR (CI)**	***n***	**AR (CI)**	***n***	**AR (CI)**	***n***	**AR (CI)**
1999	42	0.15 (0.10–0.21)	28	0.2 (0.13–0.29)	14	0.11 (0.06–0.19)	60	0.16 (0.12–0.22)	39	0.24 (0.16–0.34)	21	0.10 (0.05–0.17)
2000	37	0.14 (0.09–0.19)	21	0.15 (0.09–0.24)	16	0.12 (0.07–0.21)	70	0.17 (0.13–0.22)	37	0.20 (0.13–0.29)	33	0.14 (0.09–0.21)
2001	37	0.12 (0.08–0.17)	22	0.16 (0.10–0.25)	15	0.08 (0.04–0.15)	52	0.13 (0.09–0.18)	24	0.14 (0.09–0.22)	28	0.11 (0.07–0.18)
2002	42	0.15 (0.11–0.21)	18	0.15 (0.09–0.23)	24	0.15 (0.09–0.24)	50	0.12 (0.09–0.17)	27	0.16 (0.10–0.24)	23	0.09 (0.05–0.15)
2003	48	0.16 (0.11–0.21)	34	0.22 (0.15–0.31)	14	0.1 (0.05–0.18)	46	0.11 (0.08–0.16)	27	0.15 (0.10–0.23)	19	0.07 (0.04–0.13)
2004	56	0.18 (0.13–0.23)	29	0.17 (0.11–0.26)	27	0.18 (0.12–0.27)	35	0.08 (0.05–0.11)	19	0.09 (0.05–0.15)	16	0.07 (0.03–0.12)
2005	64	0.19 (0.15–0.25)	32	0.19 (0.13–0.28)	32	0.19 (0.13–0.28)	49	0.11 (0.08–0.15)	23	0.11 (0.07–0.17)	26	0.11 (0.07–0.17)
2006	39	0.13 (0.09–0.18)	17	0.12 (0.07–0.19)	22	0.14 (0.08–0.22)	51	0.12 (0.08–0.16)	32	0.17 (0.11–0.24)	19	0.07 (0.04–0.13)
2007	61	0.16 (0.12–0.21)	35	0.19 (0.13–0.27)	26	0.13 (0.08–0.20)	39	0.09 (0.06–0.13)	22	0.12 (0.07–0.18)	17	0.06 (0.03–0.11)
2008	58	0.15 (0.11–0.20)	33	0.17 (0.11–0.24)	25	0.13 (0.08–0.20)	44	0.11 (0.08–0.15)	16	0.08 (0.05–0.14)	28	0.13 (0.08–0.20)
2009	46	0.12 (0.09–0.16)	19	0.11 (0.06–0.17)	27	0.13 (0.08–0.20)	39	0.07 (0.05–0.10)	16	0.06 (0.03–0.11)	23	0.08 (0.05–0.13)
2010	53	0.13 (0.10–0.18)	24	0.12 (0.07–0.18)	29	0.15 (0.10–0.23)	61	0.13 (0.09–0.17)	31	0.15 (0.10–0.22)	30	0.10 (0.06–0.16)
2011	68	0.15 (0.12–0.20)	36	0.18 (0.12–0.25)	32	0.12 (0.08–0.19)	44	0.08 (0.06–0.12)	22	0.09 (0.05–0.14)	22	0.08 (0.04–0.13)
2012	56	0.12 (0.09–0.16)	29	0.13 (0.08–0.19)	27	0.12 (0.07–0.18)	50	0.07 (0.05–0.10)	25	0.09 (0.05–0.14)	25	0.06 (0.04–0.10)
2013	46	0.09 (0.06–0.13)	25	0.1 (0.06–0.15)	21	0.09 (0.05–0.15)	63	0.11 (0.08–0.14)	31	0.13 (0.08–0.19)	32	0.09 (0.05–0.14)
APC	−1.49 (*p* = 0.20)		−2.61 (*p* = 0.10)		−0.16 (*p* = 0.90)		−3.96 (*p* < 0.001)		−5.10 (*p* < 0.001)		−2.08 (*p* = 0.20)	
**Year**	**Rare CAs of the Musculoskeletal System**						**Diaphragmatic Hernia**					
	**Both Sexes**		**Men**		**Women**		**Both Sexes**		**Men**		**Women**	
	***n***	**AR (CI)**	***n***	**AR (CI)**	***n***	**AR (CI)**	***n***	**AR (CI)**	***n***	**AR (CI)**	***n***	**AR (CI)**
1999	61	0.26 (0.20–0.34)	34	0.29 (0.20–0.41)	27	0.23 (0.15–0.34)	32	0.15 (0.10–0.21)	19	0.18 (0.11–0.27)	13	0.13 (0.07–0.22)
2000	59	0.25 (0.19–0.32)	27	0.23 (0.15–0.34)	32	0.26 (0.18–0.38)	26	0.12 (0.08–0.18)	16	0.15 (0.08–0.24)	10	0.10 (0.05–0.18)
2001	49	0.21 (0.15–0.28)	31	0.26 (0.17–0.37)	18	0.15 (0.09–0.25)	29	0.13 (0.09–0.19)	19	0.17 (0.10–0.27)	10	0.10 (0.05–0.18)
2002	51	0.21 (0.15–0.27)	25	0.20 (0.13–0.30)	26	0.21 (0.14–0.32)	22	0.10 (0.06–0.15)	14	0.12 (0.07–0.20)	8	0.07 (0.03–0.14)
2003	67	0.23 (0.18–0.30)	35	0.24 (0.17–0.34)	32	0.22 (0.15–0.32)	30	0.13 (0.09–0.18)	17	0.14 (0.08–0.22)	13	0.11 (0.06–0.19)
2004	40	0.13 (0.09–0.19)	22	0.16 (0.10–0.25)	18	0.11 (0.06–0.18)	13	0.05 (0.03–0.09)	9	0.07 (0.03–0.13)	4	0.03 (0.01–0.09)
2005	46	0.15 (0.11–0.21)	19	0.12 (0.07–0.19)	27	0.19 (0.12–0.28)	23	0.09 (0.05–0.13)	12	0.08 (0.04–0.15)	11	0.09 (0.04–0.16)
2006	50	0.17 (0.12–0.22)	35	0.23 (0.16–0.33)	15	0.10 (0.06–0.17)	25	0.09 (0.06–0.14)	17	0.12 (0.07–0.19)	8	0.06 (0.03–0.12)
2007	43	0.14 (0.10–0.19)	25	0.16 (0.10–0.24)	18	0.12 (0.07–0.20)	18	0.06 (0.04–0.10)	9	0.06 (0.03–0.12)	9	0.07 (0.03–0.13)
2008	56	0.18 (0.13–0.23)	28	0.17 (0.11–0.25)	28	0.18 (0.12–0.27)	25	0.09 (0.06–0.13)	11	0.07 (0.04–0.13)	14	0.10 (0.05–0.17)
2009	40	0.12 (0.08–0.16)	21	0.12 (0.07–0.19)	19	0.11 (0.06–0.17)	16	0.05 (0.03–0.08)	8	0.05 (0.02–0.10)	8	0.05 (0.02–0.10)
2010	47	0.14 (0.10–0.18)	28	0.17 (0.11–0.24)	19	0.10 (0.06–0.17)	26	0.08 (0.06–0.12)	17	0.11 (0.06–0.17)	9	0.06 (0.03–0.11)
2011	62	0.18 (0.14–0.23)	28	0.16 (0.11–0.23)	34	0.20 (0.14–0.28)	25	0.08 (0.05–0.12)	12	0.07 (0.04–0.13)	13	0.09 (0.05–0.15)
2012	28	0.08 (0.06–0.12)	10	0.06 (0.03–0.11)	18	0.11 (0.06–0.18)	11	0.04 (0.02–0.06)	3	0.02 (0.00–0.05)	8	0.05 (0.02–0.11)
2013	29	0.08 (0.06–0.12)	11	0.07 (0.03–0.12)	18	0.10 (0.06–0.17)	18	0.06 (0.04–0.09)	8	0.05 (0.02–0.10)	10	0.07 (0.03–0.13)
APC	−5.75 (*p* < 0.001)		−6.48 (*p* < 0.001)		−4.76 (*p* < 0.001)		−6.45 (*p* < 0.001)		−8.09 (*p* < 0.001)		−3.85 (*p* < 0.001)	
**Year**	**Other Rare Congenital Malformations**						**Rare Chromosomal Abnormalities, Not Elsewhere Classified**					
	**Both Sexes**		**Men**		**Women**		**Both Sexes**		**Men**		**Women**	
	***n***	**AR (CI)**	***n***	**AR (CI)**	***n***	**AR (CI)**	***n***	**AR (CI)**	***n***	**AR (CI)**	***n***	**AR (CI)**
1999	158	0.63 (0.53–0.74)	87	0.68 (0.54–0.85)	71	0.58 (0.45–0.74)	157	0.55 (0.46–0.55)	64	0.55 (0.46–0.65)	93	0.67 (0.53–0.83)
2000	168	0.64 (0.55–0.75)	87	0.67 (0.53–0.83)	81	0.62 (0.48–0.78)	119	0.42 (0.35–0.51)	55	0.42 (0.35–0.51)	64	0.47 (0.36–0.61)
2001	154	0.61 (0.51–0.71)	89	0.68 (0.54–0.85)	65	0.53 (0.40–0.68)	116	0.38 (0.31–0.46)	47	0.38 (0.31–0.46)	69	0.47 (0.36–0.61)
2002	158	0.57 (0.48–0.68)	84	0.62 (0.49–0.78)	74	0.52 (0.40–0.66)	123	0.4 (0.33–0.49)	51	0.4 (0.33–0.49)	72	0.48 (0.37–0.62)
2003	136	0.47 (0.39–0.56)	84	0.57 (0.45–0.71)	52	0.36 (0.26–0.48)	163	0.51 (0.43–0.60)	63	0.51 (0.43–0.60)	100	0.67 (0.54–0.82)
2004	123	0.41 (0.34–0.50)	67	0.44 (0.34–0.57)	56	0.38 (0.28–0.50)	171	0.5 (0.43–0.59)	80	0.5 (0.43–0.59)	91	0.55 (0.44–0.68)
2005	143	0.44 (0.37–0.53)	77	0.46 (0.36–0.58)	66	0.43 (0.33–0.55)	176	0.5 (0.43–0.59)	78	0.5 (0.43–0.59)	98	0.57 (0.54–0.70)
2006	130	0.42 (0.34–0.50)	73	0.46 (0.36–0.58)	57	0.37 (0.28–0.49)	148	0.39 (0.33–0.46)	68	0.39 (0.33–0.46)	80	0.45 (0.35–0.56)
2007	122	0.36 (0.30–0.43)	70	0.41 (0.32–0.53)	52	0.3 (0.22–0.40)	161	0.41 (0.35–0.48)	85	0.41 (0.35–0.48)	76	0.41 (0.32–0.52)
2008	136	0.38 (0.32–0.46)	70	0.39 (0.31–0.50)	66	0.37 (0.28–0.48)	146	0.36 (0.30–0.43)	69	0.33 (0.26–0.42)	77	0.39 (0.31–0.50)
2009	126	0.35 (0.29–0.42)	65	0.34 (0.26–0.44)	61	0.35 (0.27–0.46)	175	0.42 (0.36–0.50)	93	0.44 (0.36–0.55)	82	0.41 (0.32–0.51)
2010	121	0.31 (0.26–0.38)	70	0.35 (0.27–0.45)	51	0.28 (0.21–0.37)	172	0.42 (0.36–0.49)	79	0.37 (0.29–0.47)	93	0.46 (0.37–0.57)
2011	117	0.32 (0.26–0.38)	59	0.32 (0.24–0.41)	58	0.32 (0.24–0.42)	164	0.39 (0.34–0.46)	78	0.36 (0.29–0.46)	86	0.43 (0.34–0.54)
2012	99	0.25 (0.21–0.31)	51	0.26 (0.19–0.35)	48	0.25 (0.18–0.34)	159	0.38 (0.32–0.44)	77	0.36 (0.28–0.45)	82	0.4 (0.32–0.51)
2013	87	0.23 (0.18–0.29)	42	0.22 (0.16–0.30)	45	0.24 (0.18–0.29)	163	0.37 (0.31–0.43)	81	0.36 (0.28–0.45)	82	0.38 (0.30–0.47)
APC	−6.66 (*p* < 0.001)		−7.19 (*p* < 0.001)		−6.00 (*p* < 0.001)		−1.77 (*p* < 0.001)		−0.16 (*p* = 0.90)		−3.05 (*p* < 0.001)	
**Year**	**Edwards Syndrome/Trisomy 18**											
	**Both Sexes**		**Men**		**Women**							
	***n***	**AR (CI)**	***n***	**AR (CI)**	***n***	**AR (CI)**						
1999	26	0.12 (0.08–0.18)	4	0.04 (0.01–0.09)	22	0.21 (0.13–0.32)						
2000	20	0.09 (0.06–0.14)	7	0.06 (0.03–0.13)	13	0.12 (0.07–0.21)						
2001	21	0.10 (0.06–0.15)	4	0.04 (0.01–0.09)	17	0.16 (0.09–0.26)						
2002	14	0.06 (0.03–0.10)	5	0.04 (0.01–0.10)	9	0.08 (0.04–0.15)						
2003	26	0.11 (0.07–0.16)	3	0.02 (0.01–0.07)	23	0.20 (0.13–0.30)						
2004	24	0.10 (0.06–0.14)	7	0.05 (0.02–0.11)	17	0.14 (0.08–0.23)						
2005	26	0.10 (0.06–0.15)	9	0.07 (0.03–0.13)	17	0.13 (0.08–0.21)						
2006	17	0.06 (0.04–0.10)	3	0.02 (0.00–0.06)	14	0.11 (0.06–0.18)						
2007	19	0.07 (0.04–0.10)	4	0.03 (0.01–0.07)	15	0.11 (0.06–0.18)						
2008	21	0.07 (0.04–0.11)	9	0.06 (0.03–0.11)	12	0.08 (0.04–0.15)						
2009	17	0.05 (0.03–0.09)	6	0.04 (0.01–0.08)	11	0.07 (0.04–0.13)						
2010	17	0.05 (0.03–0.09)	3	0.02 (0.00–0.06)	14	0.09 (0.05–0.16)						
2011	20	0.06 (0.04–0.10)	8	0.05 (0.02–0.10)	12	0.08 (0.04–0.14)						
2012	9	0.03 (0.01–0.06)	4	0.03 (0.01–0.06)	5	0.03 (0.01–0.08)						
2013	11	0.04 (0.02–0.06)	5	0.03 (0.01–0.07)	6	0.04 (0.01–0.09)						
APC	−6.60 (*p* < 0.001)		−2.44 (*p* = 0.30)		−8.24 (*p* < 0.001)							

**Table 3:** Standardised mortality ratio 1999–2013 (95% CI) for rare congenital anomalies, by sex. Only districts with statistically significant values are shown.

Standardised Mortality Ratio for Rare Congenital Anomalies, 1999–2013 (95% CI)					
District	Province	Location	Both Sexes	Men	Women
Significantly Lower than the Expected for the Country as Reference					
Marquesado	Alicante	E	0.69 (0.48–0.97)	–	–
Central	Alicante	E	0.76 (0.64–0.90)	0.78 (0.62–0.98)	0.74 (0.57–0.94)
Meridional	Alicante	E	0.84 (0.71–0.98)	0.80 (0.63–0.99)	–
Badajoz	Badajoz	W	0.68 (0.47–0.94)	0.58 (0.33–0.95)	–
Ibiza	Baleares	E *	–	–	0.42 (0.17–0.86)
Mallorca	Baleares	E *	0.71 (0.61–0.83)	0.66 (0.53–0.82)	0.77 (0.62–0.95)
Osona	Barcelona	NE	–	0.57 (0.32–0.94)	–
Penedès	Barcelona	NE	0.76 (0.58–0.98)	–	0.62 (0.39–0.94)
Vallès Oriental	Barcelona	NE	0.76 (0.61–0.94)	–	0.69 (0.48–0.95)
Vallès Occidental	Barcelona	NE	0.71 (0.62–0.82)	0.68 (0.56–0.83)	0.75 (0.61–0.92)
Litoral del Norte	Castellón	E	–	0.42 (0.13–0.97)	–
Cerdanya	Girona	NE	0.00 (0.00–0.87)	–	–
La Selva	Girona	NE	0.63 (0.43–0.90)	0.58 (0.32–0.96)	–
Bajo Cinca	Huesca	N	–	0.00 (0.00–0.96)	–
Costa	Lugo	NW	–	–	0.22 (0.02–0.79)
Sur Occidental	Madrid	C	0.61 (0.52–0.72)	0.62 (0.49–0.77)	0.61 (0.47–0.78)
Litoral	Pontevedra	NW	0.76 (0.65–0.89)	0.76 (0.60–0.94)	0.78 (0.61–0.97)
Costera	Cantabria	N	0.62 (0.50–0.76)	0.61 (0.45–0.81)	0.63 (0.46–0.85)
Sagra–Toledo	Toledo	C	0.71 (0.53–0.92)	0.60 (0.39–0.88)	–
Riberas del Júcar	Valencia	E	–	–	0.67 (0.44–0.99)
**Significantly Higher than the Expected for the Country as Reference**					
Campo de Níjar y Bajo Andarax	Almería	SE	1.25 (1.02–1.52)	–	1.40 (1.04–1.83)
Ávila	Avila	C	–	1.85 (1.05–3.00)	–
Don Benito	Badajoz	W	1.43 (1.02–1.94)	–	–
Osona	Barcelona	NE	–	–	1.48 (1.02–2.07)
Arlanzón	Burgos	N	–	1.40 (1.01–1.88)	–
Caceres	Caceres	W	1.42 (1.02–1.86)	1.82 (1.28–2.52)	1.44 (1.14–1.80)
Campiña de Cádiz	Cadiz	SW	1.20 (1.01–1.42)	–	–
Los Pedroches	Cordoba	S	1.47 (1.01–2.07)	–	–
Campiña Baja	Cordoba	S	1.29 (1.11–1.50)	–	1.35 (1.07–1.67)
Las Colonias	Cordoba	S	1.95 (1.05–3.00)	2.13 (1.02–3.92)	–
Campiña Alta	Cordoba	S	1.33 (1.04–1.67)	–	1.73 (1.26–2.31)
Serranía Baja	Cuenca	C	–	3.63 (1.17–8.47)	–
La Vega	Granada	S	1.32 (1.15–1.51)	1.29 (1.06–1.56)	1.36 (1.11–1.65)
Guadix	Granada	S	1.77 (1.11–2.67)	–	–
Montefrío	Granada	S	–	–	3.56 (1.77–6.36)
Sierra	Huelva	SW	–	–	2.16 (1.08–3.87)
La Loma	Jaén	S	1.44 (1.03–1.96)	–	1.85 (1.19–2.16)
Campiña del Sur	Jaén	S	1.66 (1.36–2.02)	1.88 (1.44–2.42)	1.41 (1.00–1.92)
Mágina	Jaén	S	–	–	2.21 (1.06–4.06)
Montaña de Riaño	León	N	2.37 (1.08–4.49)	–	–
Guadalhorce	Malaga	S	1.31 (1.19–1.43)	1.34 (1.18–1.52)	1.27 (1.11–1.45)
Vélez–Málaga	Malaga	S	1.40 (1.10–1.75)	1.51 (1.10–2.02)	–
Río Segura	Murcia	SE	1.22 (1.07–1.37)	1.31 (1.11–1.53)	–
Suroeste y Valle del Guadalentín	Murcia	SE	1.31 (1.05–1.60)	1.41 (1.06–1.84)	–
Campo de Cartagena	Murcia	SE	1.59 (1.36–1.84)	1.68 (1.37–2.04)	1.48 (1.16–1.85)
Grado	Asturias	N	–	–	2.33 (1.16–4.17)
Oviedo	Asturias	N	1.40 (1.17–1.66)	1.55 (1.22–1.94)	–
Gran Canaria	Las Palmas	SW *	1.34 (1.20–1.49)	1.33 (1.14–1.54)	1.36 (1.15–1.59)
Sur de Tenerife	Tenerife	SW *	1.30 (1.11–1.51)	1.32 (1.06–1.62)	1.28 (1.01–1.60)
Sierra Norte	Seville	SW	1.77 (1.22–2.49)	2.40 (1.54–3.57)	–
La Vega	Seville	SW	1.27 (1.15–1.39)	1.29 (1.14–1.47)	1.24 (1.07–1.42)
Ceuta	Ceuta	S	1.61 (1.18–2.14)	1.97 (1.34–2.80)	–

* Island territories (Canary Islands and Balearic Islands). C = Centre; E = east; N = north; NE = north-east; NW = north-west; S = south; SE = south-east; SW = south-west; W = west; Dashes (–) represent not significant standardised mortality ratio for that category.

## Supplementary Materials

The following are available online at http://www.mdpi.com/1660-4601/15/8/1715/s1, Table S1: ICD–10 codes corresponding to rare congenital anomalies (low–prevalence congenital anomalies).
