# Supplementary material for: A Nationwide Registry-Based Study on Mortality Due to Rare Congenital Anomalies
Source: Int J Environ Res Public Health. 2018 Aug 10;15(8):1715. doi: 10.3390/ijerph15081715 (PMC6121521; doi:10.3390/ijerph15081715)
Supplement: Supplementary file 1 [file ijerph-15-01715-s001.pdf]

International Journal of
Environmental Research and Public Health

MDPI

Table S1. ICD-10 codes corresponding to rare congenital anomalies (low-prevalence congenital anomalies).

RARE CONGENITAL MALFORMATIONS, DEFORMATIONS AND CHROMOSOMAL ABNORMALITIES
**Congenital malformations of the nervous system**
Q00, Q00.0, Q00.1, Q00.2, Q01, Q01.0, Q01.1, Q01.2, Q01.8, Q01.9, Q02, Q03, Q03.0, Q03.1, Q03.8, Q03.9, Q04, Q04.0, Q04.1, Q04.2, Q04.3, Q04.4, Q04.5, Q04.6, Q04.8, Q04.9, Q05, Q05.0, Q05.1, Q05.2, Q05.3, Q05.4, Q05.5, Q05.6, Q05.7, Q05.8, Q05.9, Q06, Q06.0, Q06.1, Q06.2, Q06.3, Q06.4, Q06.8, Q06.9, Q07, Q07.0, Q07.8, Q07.9
**Congenital malformations of eye, ear, face and neck**
Q10.0, Q10.1, Q10.2, Q10.4, Q10.5, Q10.6, Q10.7, Q11, Q11.0, Q11.1, Q11.2, Q11.3, Q12.0, Q12.1, Q12.2, Q12.3, Q12.4, Q12.8, Q12.9, Q13, Q13.0, Q13.1, Q13.2, Q13.3, Q13.4, Q13.5, Q13.8, Q13.9, Q14, Q14.0, Q14.1, Q14.2, Q14.3, Q14.8, Q14.9, Q15, Q15.0, Q15.8, Q15.9, Q16, Q16.0, Q16.1, Q16.2, Q16.3, Q16.4, Q16.5, Q16.9, Q17.8, Q17.9, Q18.0, Q18.1, Q18.2, Q18.3, Q18.8
**Congenital malformations of the circulatory system**
Q20, Q20.0, Q20.1, Q20.2, Q20.3, Q20.4, Q20.5, Q20.6, Q20.8, Q20.9, Q21.2, Q21.3, Q21.4, Q21.8, Q21.9, Q22, Q22.0, Q22.1, Q22.2, Q22.3, Q22.4, Q22.5, Q22.6, Q22.8, Q22.9, Q23, Q23.0, Q23.1, Q23.2, Q23.3, Q23.4, Q23.8, Q23.9, Q24, Q24.0, Q24.1, Q24.2, Q24.3, Q24.4, Q24.5, Q24.6, Q24.8, Q24.9, Q25, Q25.0, Q25.1, Q25.2, Q25.3, Q25.4, Q25.5, Q25.6, Q25.7, Q25.8, Q25.9, Q26, Q26.0, Q26.1, Q26.2, Q26.3, Q26.4, Q26.5, Q26.6, Q26.8, Q26.9, Q27, Q27.1, Q27.2, Q27.3, Q27.4, Q27.8, Q27.9, Q28, Q28.0, Q28.1, Q28.2, Q28.3, Q28.8, Q28.9
**Congenital malformations of the respiratory system**
Q30, Q30.0, Q30.1, Q30.2, Q30.3, Q30.8, Q30.9, Q31, Q31.0, Q31.1, Q31.2, Q31.3, Q31.5, Q31.8, Q31.9, Q32, Q32.0, Q32.1, Q32.2, Q32.3, Q32.4, Q33, Q33.0, Q33.1, Q33.2, Q33.3, Q33.4, Q33.5, Q33.6, Q33.8, Q33.9, Q34, Q34.0, Q34.1, Q34.8, Q34.9
**Cleft lip and cleft palate**
Q35.1, Q35.3, Q35.5, Q35.7, Q35.9, Q36, Q36.0, Q36.1, Q36.9, Q37, Q37.0, Q37.1, Q37.2, Q37.3, Q37.4, Q37.5, Q37.8, Q37.9
**Other congenital malformations of the digestive system**
Q38, Q38.0, Q38.1, Q38.3, Q38.4, Q38.5, Q38.6, Q38.7, Q39, Q39.0, Q39.1, Q39.2, Q39.3, Q39.4, Q39.5, Q39.6, Q39.8, Q39.9, Q40, Q40.0, Q40.1, Q40.2, Q40.3, Q40.8, Q40.9, Q41, Q41.0, Q41.1, Q41.2, Q41.8, Q41.9, Q42, Q42.0, Q42.1, Q42.2, Q42.3, Q42.8, Q42.9, Q43, Q43.0, Q43.1, Q43.2, Q43.3, Q43.4, Q43.5, Q43.6, Q43.7, Q43.8, Q43.9, Q44, Q44.0, Q44.1, Q44.2, Q44.3, Q44.4, Q44.5, Q44.6, Q44.7, Q45.0, Q45.1, Q45.2, Q45.3, Q45.8, Q45.9
**Congenital malformations of genital organs**
Q50, Q50.0, Q50.1, Q50.2, Q50.3, Q50.4, Q50.5, Q50.6, Q51, Q51.0, Q51.1, Q51.2, Q51.3, Q51.4, Q51.5, Q51.6, Q51.7, Q51.8, Q51.9, Q52, Q52.0, Q52.1, Q52.2, Q52.3, Q52.4, Q52.5, Q52.6, Q52.7, Q52.8, Q52.9, Q53.0, Q54.1, Q54.2, Q54.3, Q54.4, Q54.8, Q54.9, Q55, Q55.0, Q55.1, Q55.2, Q55.3, Q55.4, Q55.5, Q55.8, Q55.9, Q56, Q56.0, Q56.1, Q56.2, Q56.3, Q56.4
**Congenital malformations of the urinary system**
Q60, Q60.0, Q60.1, Q60.2, Q60.3, Q60.4, Q60.5, Q60.6, Q61.0, Q61.1, Q61.2, Q61.3, Q61.4, Q61.5, Q61.8, Q61.9, Q63, Q63.0, Q63.1, Q63.2, Q63.3, Q63.8, Q63.9, Q64, Q64.0, Q64.1, Q64.2, Q64.3, Q64.4, Q64.5, Q64.6, Q64.7, Q64.8, Q64.9
**Congenital malformations and deformations of the musculoskeletal system**
Q66.1, Q66.2, Q66.3, Q66.9, Q67, Q67.0, Q67.1, Q67.2, Q67.3, Q67.4, Q67.5, Q67.8, Q68, Q68.0, Q68.1, Q68.2, Q68.3, Q68.4, Q68.5, Q68.8, Q69.0, Q69.1, Q69.2, Q69.9, Q70.0, Q70.1, Q70.2, Q70.3, Q70.4, Q70.9, Q71, Q71.0, Q71.1, Q71.2, Q71.3, Q71.4, Q71.5, Q71.6, Q71.8, Q71.9, Q72, Q72.0, Q72.1, Q72.2, Q72.3, Q72.4, Q72.5, Q72.6, Q72.7, Q72.8, Q72.9, Q73, Q73.0, Q73.1, Q73.8, Q74, Q74.0, Q74.1, Q74.2, Q74.3, Q74.8, Q74.9, Q75.0, Q75.1, Q75.2, Q75.3, Q75.4, Q75.5, Q75.8, Q75.9, Q76, Q76.1, Q76.2, Q76.3, Q76.4, Q76.5, Q76.6, Q76.7, Q76.8, Q76.9, Q77, Q77.0, Q77.1, Q77.2, Q77.3, Q77.4, Q77.5, Q77.6, Q77.7, Q77.8, Q77.9, Q78, Q78.0, Q78.1, Q78.2, Q78.3, Q78.4, Q78.5, Q78.6, Q78.8, Q78.9, Q79.0, Q79.1, Q79.2, Q79.3, Q79.4, Q79.5, Q79.6, Q79.8, Q79.9
**Other congenital malformations**
Q80, Q80.0, Q80.1, Q80.2, Q80.3, Q80.4, Q80.8, Q80.9, Q81, Q81.0, Q81.1, Q81.2, Q81.8, Q81.9, Q82, Q82.0, Q82.1, Q82.2, Q82.3, Q82.4, Q82.5, Q82.9, Q83, Q83.0, Q83.1, Q83.2, Q83.3, Q83.8, Q83.9, Q84.0, Q84.1, Q84.3, Q84.4, Q84.5, Q84.8, Q84.9, Q85, Q85.0, Q85.1, Q85.8, Q85.9, Q86, Q86.0, Q86.1, Q86.2, Q86.8, Q87, Q87.0, Q87.1, Q87.2, Q87.3, Q87.4, 87.5, Q87.8, Q89, Q89.0, Q89.1, Q89.2, Q89.3, Q89.4, Q89.7, Q89.8, Q89.9
**Chromosomal abnormalities, not elsewhere classified**

Q90.1, Q90.2, Q90.9, Q91, Q91.0, Q91.1, Q91.2, Q91.3, Q91.4, Q91.5, Q91.6, Q91.7, Q92, Q92.0, Q92.1, Q92.2, Q92.3, Q92.4, Q92.5, Q92.6, Q92.7, Q92.8, Q92.9, Q93, Q93.0, Q93.1, Q93.2, Q93.3, Q93.4, Q93.5, Q93.6, Q93.7, Q93.8, Q93.9, Q95, Q95.0, Q95.1, Q95.2, Q95.3, Q95.4, Q95.5, Q95.8, Q95.9, Q96, Q96.0, Q96.1, Q96.2, Q96.3, Q96.4, Q96.8, Q96.9, Q97, Q97.0, Q97.1, Q97.2, Q97.3, Q97.8, Q97.9, Q98, Q98.0, Q98.1, Q98.2, Q98.3, Q98.4, Q98.5, Q98.6, Q98.7, Q98.8, Q98.9, Q99, Q99.0, Q99.1, Q99.2, Q99.8, Q99.9

Note: ICD-10 codes corresponding to rare diseases of the following subgroups *Congenital malformations of eye, ear, face and neck*, *Cleft lip and cleft palate* and *Congenital malformations of genital organs* are displayed in this table, but not included in the time trend analysis by type of CA due to the very low number of deceases attributed to them (6 deaths in 15 years).
